# Stürze und Polypharmazie – Häufigkeiten und Ansatzpunkte für Prävention

**DOI:** 10.1007/s00103-026-04290-y

**Published:** 2026-08-14

**Authors:** Judith Fuchs, Giselle Sarganas, Dinara Yessimova, Eva Grill

**Affiliations:** 1 Abteilung für Epidemiologie und Gesundheitsmonitoring, FG 25, Robert Koch-Institut, Nordufer 20, 13353 Berlin, Deutschland; 2 Institut für Medizinische Informationsverarbeitung, Biometrie und Epidemiologie, Deutsches Schwindel- und Gleichgewichtszentrum (DSGZ), Medizinische Fakultät, Ludwig-Maximilians-Universität München, München, Deutschland

**Keywords:** Hochaltrige, Deutschland, Längsschnittanalyse, Medikamenteneinnahme, Risikofaktoren, Old age, Germany, Longitudinal analysis, Medicine use, Risk factors

## Abstract

**Einleitung:**

Angesichts des demografischen Wandels gewinnt die Prävention von Stürzen bei Hochaltrigen zunehmend an Bedeutung. Der Beitrag beschreibt das Sturzgeschehen bei Personen ab 80 Jahren in Deutschland und untersucht relevante Risikofaktoren mit besonderem Fokus auf den Arzneimittelgebrauch.

**Methoden:**

Grundlage sind Daten der epidemiologischen Längsschnittstudie Gesundheit 65+ zur gesundheitlichen Lage älterer Menschen in Deutschland. Analysiert wurden die 12-Monats-Sturzprävalenz bei Personen ab 80 Jahren sowie Risikofaktoren wie vorangegangene Stürze, Polypharmazie (dauerhafte Einnahme von ≥ 5 Arzneimitteln), sturzbegünstigende Arzneimittel (Fall-Risk-Increasing Drugs, FRIDs), körperliche und kognitive Einschränkungen sowie soziodemografische Merkmale.

**Ergebnisse:**

Die Studienpopulation umfasste 1742 Personen ab 80 Jahren. Für die Medikamentenanalyse standen Daten von 742 Teilnehmenden zur Verfügung. 38,1 % berichteten bereits vor Studienbeginn mindestens einen Sturz. Innerhalb von 12 Monaten nach der Basisbefragung stürzten 46,7 % der Teilnehmenden. Die Polypharmazie-Prävalenz lag bei 48,7 % und 59,2 % der Teilnehmenden nahmen mindestens ein FRID ein. Im adjustierten Regressionsmodell waren Harninkontinenz, Sehbeeinträchtigung und depressive Symptomatik mit einer höheren Sturzwahrscheinlichkeit verbunden. Polypharmazie und FRIDs zeigten ebenfalls eine Assoziation mit Stürzen, die nach Adjustierung nicht statistisch signifikant war.

**Diskussion:**

Die hohe Sturzprävalenz bei Menschen ab 80 Jahren verdeutlicht den Bedarf an wirksamerer Sturzprävention in Deutschland. Relevante Ansatzpunkte für Präventionsmaßnahmen sind insbesondere Mobilitätseinschränkungen, frühere Stürze, Harninkontinenz, Sehbeeinträchtigung, depressive Symptomatik sowie Polypharmazie und FRIDs.

## Abstract (en)

**Introduction:**

In light of demographic changes, preventing falls among very old people is becoming increasingly important. This article examines the incidence of falls among people aged 80 years and over in Germany, focusing particularly on the impact of medication use.

**Methods:**

The analysis is based on data from the Gesundheit 65+ longitudinal epidemiological study of the health of people aged 65 and over in Germany. The analysis examined the prevalence of falls over a 12-month period among people aged 80 and over, as well as risk factors such as previous falls, polypharmacy (the long-term use of five or more medications), the use of fall-risk-increasing drugs (FRIDs), physical and cognitive limitations, and sociodemographic factors.

**Results:**

The study population comprised 1742 individuals aged 80 and over. Data from 742 participants were available for the medication analysis. At baseline, 38.1% of participants reported at least one fall prior to the start of the study. Within the 12-month period following the baseline survey, 46.7% of participants experienced a fall. The prevalence of polypharmacy was 48.7%, and 59.2% of participants were taking at least one FRID. In the adjusted regression model, urinary incontinence, visual impairment, and depressive symptoms were associated with an elevated probability of falling. Polypharmacy and FRIDs were also associated with falls, but this association was not statistically significant after adjustment.

**Discussion:**

The high prevalence of falls among people aged 80 and over highlights the need for more effective fall prevention measures in Germany. Key areas for preventive measures include mobility limitations, previous falls, urinary incontinence, visual impairment, depressive symptoms, polypharmacy, and FRIDs.

## Einleitung

Ein Sturz stellt über den gesamten Lebensverlauf, insbesondere im hohen Alter, ein Ereignis mit potenziell schwerwiegenden Folgen dar. Balzer et al. [1] weisen für die Definition auf die Empfehlung der Konsensuskonferenz des Prevention of Falls Network Europe (ProFaNE; [2]) hin, nach der ein Sturz definiert ist als: „an unexpected event in which the participants come to rest on the ground, floor, or lower level“, die auch von der Weltgesundheitsorganisation (WHO) verwendet wird [3]. Diese Definition wird im Folgenden den Analysen zugrunde gelegt.

Die durchschnittliche Sturzhäufigkeit bei in Privathaushalten lebenden Personen ab 65 Jahren in Europa liegt bei über 30 % [4–6]. Zwischen 20 % und 30 % der Gestürzten erleiden Verletzungen, die ihre Mobilität und Unabhängigkeit einschränken und das Risiko eines vorzeitigen Todes erhöhen [7]. Für Deutschland wird bei zu Hause lebenden Personen ein Anteil von 47 % für Stürze mit Verletzungsfolgen geschätzt [1]. Mit zunehmendem Alter steigt die Sturzprävalenz [6, 8, 9]; bundesweite Daten aus dem Jahr 2022 für Privathaushalte zeigen, dass Personen ab 80 Jahren deutlich häufiger stürzen (33,5 %) als Personen im Alter zwischen 65 und 79 Jahren (19,2 %; [10]).

Im Jahr 2024 betrug der Anteil der Über-80-Jährigen an der Bevölkerung 7,2 %. Er wird bis 2050 voraussichtlich auf bis zu 12 % ansteigen [11]. Angesichts der Tatsache, dass in Zukunft immer mehr ältere und hochaltrige Menschen in Deutschland leben werden, spielen für die Gesundheit und die Erhaltung von Selbstständigkeit Stürze und die zugrunde liegenden Risikofaktoren eine wichtige Rolle.

Die Risikofaktoren für Stürze ordnet die WHO [3] verschiedenen Bereichen zu: (1) *biologische Faktoren*, die mit Krankheiten und dem natürlichen Alterungsprozess zusammenhängen, d. h. nicht veränderbare Faktoren wie Alter, Geschlecht und ethnische Zugehörigkeit, die in Zusammenhang mit altersbedingten Veränderungen wie dem Rückgang körperlicher und kognitiver Fähigkeiten und der Zunahme von chronischen Erkrankungen stehen, (2) *Verhaltensfaktoren*, d. h. potenziell veränderbare Faktoren, die menschliche Handlungen, Emotionen oder tägliche Entscheidungen betreffen, etwa riskante Verhaltensweisen wie die Einnahme mehrerer Arzneimittel, übermäßiger Alkoholkonsum und Bewegungsmangel, die Verwendung von ungeeigneten Hilfsmitteln sowie Sturzangst, (3) *soziale und wirtschaftliche Faktoren *wie geringes Einkommen, geringer Bildungsstand, unzureichende Wohnverhältnisse, mangelnde soziale Interaktion (Einsamkeit) oder eingeschränkter Zugang zu Gesundheits- und Sozialversorgung, (4) *Umweltfaktoren*, d. h. Faktoren, die im Zusammenspiel von körperlicher Verfassung und Umgebung relevant werden, wie Gefahren im Haushalt (z. B. lose Teppiche, unzureichende Beleuchtung) und in der Wohnumgebung (z. B. unebene Gehwege) sowie klimatische Faktoren (Eis, Hitze). Zudem erhöhen vorangegangene Stürze das Risiko für weitere Stürze [5, 9, 12].

Nach einer aktuellen Literaturübersicht sind die 10 häufigsten Prädiktoren für Stürze: Einschränkungen beim Gleichgewicht, in der Kognition, beim Gehen, in der körperlichen Funktion und bei körperlicher Aktivität sowie die Einnahme von Arzneimitteln, chronische Erkrankungen, eingeschränkte Dual-Task-Fähigkeit, Gebrechlichkeit und verminderte Kraft [13]. Stürze bei Hochaltrigen sind selten auf eine einzelne Ursache zurückzuführen, sondern resultieren in der Regel aus der Wechselwirkung zwischen individuellen Prädispositionen und akuten auslösenden Faktoren [6, 8]. Trotz evidenzbasierter Präventionsansätze ist die Sturzprävalenz in Europa bislang nicht gesunken [4], was auf Umsetzungsdefizite hindeutet.

Polypharmazie, die dauerhafte Einnahme von 5 oder mehr Arzneimitteln, ist im höheren Lebensalter weitverbreitet und nimmt mit dem Alter zu. Auf Grundlage von Daten der gesetzlichen Krankenversicherung (GKV) lag 2023 die Prävalenz von Polypharmazie bei 58–62 % der 80- bis Unter-85-Jährigen [14]. Hyperpolypharmazie, die Einnahme von 10 oder mehr Wirkstoffen, betrifft bei den 85- bis 90-Jährigen nahezu 20 % [15–17]. Altersbedingte pharmakokinetische und -dynamische Veränderungen erhöhen das Risiko unerwünschter Arzneimittelwirkungen (UAW) und Interaktionen zwischen Arzneistoffen [18–20]. Zusätzlich können sich Arzneimittel, die zur Therapie einer Erkrankung eingesetzt werden, negativ auf andere Erkrankungen auswirken oder mit anderen verordneten Wirkstoffen interagieren (Wechselwirkungen; [19, 21]). UAW sind ein wesentlicher Faktor für ungeplante Krankenhausaufnahmen, insbesondere bei Patientinnen und Patienten mit Polypharmazie. Durch UAW-bedingte Stürze kommt es zu einer kumulativen Inzidenz von 209,6 Krankenhauseinweisungen pro 10.000 Bewohnerinnen und Bewohnern [22]. Ein linearer Zusammenhang zwischen Sturzrisiko und der Anzahl der eingenommenen Arzneimittel ist gut belegt. Dabei steigt das Sturzrisiko mit jedem zusätzlich täglich eingenommenen Wirkstoff signifikant an [23, 24]. So weisen Personen über 60 Jahren mit Polypharmazie eine um 21 % höhere Sturzrate auf als gleichaltrige Personen mit geringerem Arzneimittelkonsum [25].

Zudem erhöhen bestimmte Wirkstoffgruppen, die unter der Bezeichnung sturzrisikosteigernde Arzneimittel bzw. Fall Risk Increasing Drugs (FRIDs) zusammengefasst werden, z. B. Antidepressiva, Benzodiazepine, Antipsychotika oder Schleifendiuretika, unabhängig von der Gesamtzahl der eingenommenen Arzneimittel, das Sturzrisiko [26–28].

Vor diesem Hintergrund analysiert der folgende Beitrag Sturzhäufigkeit und Arzneimitteleinnahme bei Personen ab 80 Jahren, um Ansatzpunkte für eine verbesserte Prävention zu identifizieren.

## Methoden

### Die Studie Gesundheit 65+

Die Studie Gesundheit 65+ war eine epidemiologische Längsschnittstudie zum Gesundheitszustand von Personen ab 65 Jahren in Deutschland, die im Zeitraum Juni 2021 bis April 2023 durchgeführt wurde. Die Responserate zur Basisbefragung nach den Richtlinien der American Association for Public Opinion Research (AAPOR; [29]) lag bei 30,9 %. Die Zielpopulation umfasste Personen ab 65 Jahren, die ihren ständigen Wohnsitz in Deutschland hatten. Personen mit unzureichenden Deutschkenntnissen sowie Personen, die vor Studienbeginn verstorben oder umgezogen bzw. nicht auffindbar waren, wurden von der Teilnahme ausgeschlossen. Die Teilnehmenden selbst oder ihre gesetzlichen Vertretungen gaben schriftlich ihr Einverständnis zur Teilnahme [30]. Personen, die selbst keine Angaben zu ihrer Gesundheit machen oder nicht an der Befragung teilnehmen konnten, hatten die Möglichkeit, über die Befragung einer Stellvertreterin oder eines Stellvertreters teilzunehmen [30].

Detaillierte Informationen zu Rekrutierung und Stichprobenziehung sind bei Fuchs et al. 2023 beschrieben [30]. Die Teilnehmenden wurden insgesamt 4‑mal befragt (Basisbefragung und 3 Nachbefragungen innerhalb von 12 Monaten) und im Rahmen der dritten Nachbefragung wurde ein Untersuchungsmodul während eines Hausbesuchs durchgeführt. An der Basisbefragung nahmen insgesamt 3694 Personen teil, an der ersten Nachbefragung 3164, an der zweiten 3111, an der dritten 2935 Personen und an der nachfolgenden Untersuchung 1493 Personen. Sämtliche Angaben zu den folgenden Variablen wurden in der Basisbefragung erhoben, mit Ausnahme der Stürze (Basisbefragung und 3 Nachbefragungen) sowie der eingenommenen Arzneimittel (Untersuchungsteil).

### Erfassung der Stürze und ihrer Risikofaktoren

Die Sturzanamnese erfolgte zur Basisbefragung und prospektiv über einen Zeitraum von 12 Monaten. Zur Basisbefragung gaben die Teilnehmenden basierend auf [2] an, ob sie innerhalb der letzten 12 Monate gefallen, gestolpert oder ausgerutscht sind, sodass sie ihr Gleichgewicht verloren haben und auf dem Boden oder einer tieferen Ebene gelandet sind. Falls dies bejaht wurde, wurde gefragt, ob dies ein-, 2‑ oder mehrmals vorgekommen ist. Stürze zur Basisbefragung wurden kategorisiert in „Keine“ (keine Stürze in den vergangenen 12 Monaten), „Ein Sturz“ und „Zwei und mehr Stürze“. Zusätzlich wurde eine dichotome Variable gebildet („Mindestens ein Sturz“ vs. „Keine“).

Stürze wurden in allen 3 Nachbefragungswellen (Wellen 1–3) erfasst, wobei Teilnehmende in jeder Welle angaben, ob und wie oft sie seit der letzten Befragung gestürzt waren. Auf Basis der Angaben aus den 3 Nachbefragungen wurden 2 Variablen gebildet. Die kategoriale Variable berücksichtigte sowohl die Anzahl der betroffenen Wellen als auch die Gesamtzahl der Stürze: „Zwei und mehr Stürze“ (bei Stürzen in mindestens 2 Wellen oder eine Gesamtzahl von mehr als einem Sturz), „Ein Sturz“ (genau ein berichteter Sturz über alle 3 Wellen hinweg) und „Keine“ (kein berichteter Sturz in allen 3 Wellen). Analog zu den Angaben aus der Basisbefragung wurde eine dichotome Variable gebildet („Mindestens ein Sturz“ vs. „Keine“).

Für die Auswahl der Sturzrisikofaktoren wurde der WHO-Bericht [3] zugrunde gelegt, der zwischen unveränderbaren biologischen Faktoren, veränderbaren verhaltensbedingten, umgebungsbedingten sowie sozioökonomischen Faktoren unterscheidet und damit Ansatzmöglichkeiten für die Diskussion von Präventionsmaßnahmen bietet.

#### Biologische Risikofaktoren

*Multimorbidität* wurde über die selbstberichtete 12-Monats-Prävalenz von 10 im European Health Interview Survey [31] aufgeführten chronischen Erkrankungen erfasst: (1) Hypertonie, (2) koronare Herzkrankheit (einschließlich Myokardinfarkt oder chronischer Beschwerden nach Myokardinfarkt sowie Angina pectoris), (3) Schlaganfall (einschließlich chronischer Beschwerden nach Schlaganfall), (4) Hypercholesterinämie, (5) Diabetes mellitus, (6) chronische Bronchitis (einschließlich chronisch obstruktive Lungenerkrankung oder Emphysem), (7) Arthrose, (8) Osteoporose, (9) Beschwerden im unteren Rücken oder sonstige chronische Rückenleiden und (10) Depression. Zusätzlich wurde (11) die Lebenszeitprävalenz von Krebserkrankungen eingeschlossen, die mit der Frage erhoben wurde, ob jemals eine ärztliche Krebsdiagnose gestellt wurde. Die Anzahl der berichteten Erkrankungen wurde summiert (Range 0 bis 11). Multimorbidität wurde als das Vorliegen von 2 oder mehr Erkrankungen definiert.

*Harninkontinenz* wurde anhand der Frage: „Hatten Sie in den letzten 12 Monaten Harninkontinenz bzw. Probleme, die Blase zu kontrollieren?“, erhoben; eine Bejahung kennzeichnete das Vorliegen von Harninkontinenz.

*Mobilitätseinschränkungen* wurden als dichotome Variable erfasst und galten als vorhanden, wenn Teilnehmende entweder Schwierigkeiten angaben, einen halben Kilometer auf ebenem Gelände ohne Gehhilfe zu gehen oder eine Treppe mit 12 Stufen zu steigen.

*Seheinschränkungen* wurden als schwerwiegend klassifiziert, wenn Teilnehmende mindestens große Schwierigkeiten beim Sehen trotz Brille oder Kontaktlinsen angaben.

*Subjektive Gedächtnisverschlechterung* wurde gemäß der AgeCoDe-Study [32] als dichotome Variable erfasst und galt als vorhanden, wenn Teilnehmende sowohl auf „Haben Sie das Gefühl, Ihr Gedächtnis wird schlechter?“ als auch auf „Macht Ihnen das Sorgen?“ mit „Ja“ antworteten.

*Depressive Symptomatik* in den vergangenen 2 Wochen wurde mit dem Patient Health Questionnaire (PHQ-2) erfasst [33]. Hier werden Beeinträchtigungen durch geringes Interesse oder Freude an Tätigkeiten sowie Niedergeschlagenheit, Schwermut oder Hoffnungslosigkeit abgefragt (Summenwert 0–6). Ein Summenwert von ≥ 3 wurde als Vorliegen depressiver Symptomatik definiert.

#### Verhaltensbedingte Risikofaktoren

*Adipositas* wurde auf Basis der selbstberichteten Angaben zu Körpergewicht und -größe gemäß WHO-Definition als Body-Mass-Index (BMI) von ≥ 30 kg/m^2^ definiert.

*Polypharmazie* wurde über die Frage: „Wie viele unterschiedliche von einer Ärztin/einem Arzt verschriebene Arzneimittel haben Sie in den letzten 7 Tagen eingenommen?“, erfasst, mit den Antwortoptionen: „Keine“, „1 bis 4 Arzneimittel“ und „5 und mehr Arzneimittel“. Polypharmazie wurde als gleichzeitige Einnahme von mindestens 5 Arzneimitteln definiert [17].

Die verwendeten Arzneimittel und speziell FRIDs wurden vertiefend im Rahmen der Untersuchung in Welle 3 erfasst. Die Teilnehmenden wurden gebeten, die Packungen aller in den vergangenen 7 Tagen eingenommenen Arzneimittel (verordnete und Selbstmedikation) bereitzulegen. Die Barcodes der Packungen wurden eingescannt und die dem Code entsprechende Pharmazentralnummer wurde gemäß der ATC-Klassifikation (Anatomical Therapeutic Chemical) kategorisiert [30].

Die Klassifizierung der FRIDs erfolgte analog STOPPFall (Screening Tool of Older Persons Prescriptions in Older Adults with High Fall Risk) auf Basis der ATC-Codes [34–36]. Sie umfassen die Klassen Benzodiazepine (N03AE, N05BA, N05CD), Antipsychotika (N05A), Benzodiazepin-verwandte Arzneimittel (N05CF), Opioide (N02A), Antidepressiva (N06A), Anticholinergika (N04A, R03BB, S01FA), Antiepileptika (N03A), Diuretika (C03, C02L), Alpha-Blocker-Antihypertensiva (C02CA), Alpha-Blocker bei Prostatahyperplasie (G04CA), zentral wirkende Antihypertensiva (C02A), Antihistaminika (R06A), Vasodilatatoren bei Herzerkrankungen (C01D) und Arzneimittel bei überaktiver Blase/Dranginkontinenz (G04BD). Die Einnahme von FRIDs wurde definiert als die Anwendung mindestens einer der aufgeführten Substanzen in den letzten 7 Tagen.

#### Sozioökonomische Risikofaktoren

Das *Bildungsniveau* wurde anhand der CASMIN-Klassifikation (Comparative Analysis of Social Mobility in Industrial Nations) anhand des jeweils höchsten schulischen und berufsbildenden Bildungsabschlusses in 3 Gruppen, niedrige (d. h. maximal Hauptschulabschluss mit und ohne beruflichen Abschluss), mittlere (d. h. mittlere Reife oder Fach‑/Hochschulreife mit und ohne beruflichen Abschluss) und höhere Bildung (d. h. Fachhoch‑/Hochschulabschluss), zusammengefasst [37].

Die *Wohnsituation* wurde als dichotome Variable „Alleinlebend“ (Ja/Nein) erfasst.

### Statistische Analyse

Zur Berücksichtigung des komplexen Stichprobendesigns wurden Gewichtungsfaktoren und Designvariablen verwendet, die Abweichungen der Teilnehmenden von der Zielpopulation der Menschen ab 65 Jahren in Deutschland zum Stichtag 31.12.2020 hinsichtlich des Geschlechts, Alters, der Region und Gemeindegröße nach BIK-10-Klassifikation [38] korrigieren. Kategoriale Variablen wurden als absolute Häufigkeiten und gewichtete Prozentwerte dargestellt. Zusammenhänge zwischen den Sturzvariablen (Stürze zur Basisbefragung und Stürze nach 12 Monaten) sowie den Kovariaten wurden mittels gewichteter Chi-Quadrat-Tests geprüft.

Die Risikofaktoren wurden in 2 komplementären Schritten untersucht: Zunächst erfolgten deskriptive und univariable (unadjustierte) Analysen, in denen die Verteilung jedes potenziellen Risikofaktors nach Sturzstatus dargestellt wurde – mit gewichteten Anteilen und Rao-Scott-Chi-Quadrat-Tests für Gruppenvergleiche. Anschließend wurden alle Risikofaktoren gemeinsam in ein multivariables, surveygewichtetes binärlogistisches Regressionsmodell aufgenommen, um adjustierte Odds Ratios zu berechnen. Für die Regressionsanalyse wurde die dichotome Variable „Stürze innerhalb von 12 Monaten nach der Basisbefragung“ verwendet („kein Sturz“ versus „mindestens ein Sturz“). Statistisch signifikante Kovariaten aus der univariaten Analyse mit einem *p*-Wert < 0,05 wurden in das Modell aufgenommen. Es wurde ein surveygewichtetes binärlogistisches Regressionsmodell (Quasi-Binomial-Familie) berechnet, das für Geschlecht, Polypharmazie (bzw. FRIDs), Multimorbidität, Harninkontinenz, Sehbeeinträchtigung, subjektive Gedächtnisverschlechterung, depressive Symptomatik, Adipositas und Bildung adjustierte. Vorangegangene Stürze und Mobilitätseinschränkungen wurden bewusst nicht in das Modell aufgenommen, da diese Variablen teilweise auf dem kausalen Pfad zwischen Arzneimitteleinnahme und Stürzen liegen und eine Überadjustierung bewirken könnten. Fehlende Werte in den Kovariablen wurden durch listenweisen Ausschluss behandelt.

Alle Analysen wurden mit R (Version 4.3.0) und RStudio (Version 2024.4.0.735) durchgeführt. Statistische Signifikanz wurde mit *p* < 0,05 definiert.

## Ergebnisse

### Stichprobenbeschreibung

Es wurden 1742 Personen im Alter von 80 Jahren oder älter zur Basiserhebung (mittleres Alter 84,6 Jahre; 61,7 % Frauen) und 1218 Personen im Alter von 65–79 Jahren (mittleres Alter 71,9; 53,1 % Frauen) in die Analysen eingeschlossen.

### Häufigkeit von Stürzen

In der Basisbefragung gaben 38,1 % der Befragten ab 80 Jahren an, mindestens einmal gestürzt zu sein (*n* = 562/1742). Innerhalb der folgenden 12 Monate lag der Anteil mit 46,7 % etwas höher (*n* = 668/1742). Signifikante Geschlechterunterschiede zeigten sich weder zur Basisbefragung noch nach 12 Monaten. Zur Basisbefragung waren 41,1 % der Frauen und 33,3 % der Männer mindestens einmal gestürzt, im Folgejahr 48,4 % der Frauen und 44,2 % der Männer.

Insgesamt war die Sturzprävalenz bei Personen ab 80 Jahren durchgängig höher als bei den 65- bis 79-Jährigen (*p* < 0,001). Dies galt sowohl zur Basisbefragung als auch nach 12 Monaten, insbesondere für wiederholte Stürze: ≥ 2 Stürze berichteten 24,3 % vs. 8,4 % zur Basisbefragung und 33,6 % vs. 10,7 % nach 12 Monaten. Entsprechend war der Anteil ohne Sturz bei den ab 80-Jährigen niedriger.

Die Polypharmazie-Prävalenz bei Personen ab 80 Jahren lag bei 48,7 %. Auch die meisten anderen Risikofaktoren traten bei Personen ab 80 Jahren häufiger auf als bei den 65- bis 79-Jährigen. Dazu zählten Multimorbidität, Harninkontinenz, Mobilitätseinschränkungen, Sehbeeinträchtigung, subjektive Gedächtnisverschlechterung und depressive Symptomatik. Zudem hatten Teilnehmende ab 80 Jahren häufiger einen niedrigeren Bildungsstand und lebten öfter allein. Alle Unterschiede waren signifikant (*p* < 0,001; Tab. 1).

**Tab. 1:** Merkmale der Stichprobe nach Altersgruppe und Geschlecht (gewichtete %, rohe *n* und *p*-Werte für Gruppenunterschiede)

	Nach Altersgruppen			Nach Geschlecht, 80 Jahre und älter		
Kategorie	65–79 Jahre (*n* = 1218)	80 Jahre und älter (*n* = 1742)	*p*-Wert (65–79 vs. 80+)	Frauen (61,7 %; *n* = 812)	Männer (38,3 %; *n* = 930)	*p*-Wert (Frauen vs. Männer)
*Stürze (zur Basisbefragung)*						
Keine	81,5 %, (*n* = 997)	61,9 %, (*n* = 1119)	< 0,001***	58,9 %, (*n* = 496)	66,7 %, (*n* = 623)	0,058
Ein Sturz	10,1 %, (*n* = 107)	13,8 %, (*n* = 235)		14,7 %, (*n* = 124)	12,4 %, (*n* = 111)	
2 und mehr Stürze	8,4 %, (*n* = 90)	24,3 %, (*n* = 327)		26,4 %, (*n* = 168)	20,9 %, (*n* = 159)	
*Stürze (nach 12 Monaten)*						
Keine	78,1 %, (*n* = 889)	53,3 %, (*n* = 887)	< 0,001***	51,6 %, (*n* = 384)	55,8 %, (*n* = 503)	0,450
Ein Sturz	11,2 %, (*n* = 119)	13,1 %, (*n* = 245)		13,4 %, (*n* = 123)	12,6 %, (*n* = 122)	
2 und mehr Stürze	10,7 %, (*n* = 119)	33,6 %, (*n* = 423)		35,0 %, (*n* = 201)	31,6 %, (*n* = 222)	
*Multimorbidität*	64,4 %, (*n* = 762)	78,6 %, (*n* = 1290)	< 0,001***	82,4 %, (*n* = 647)	72,5 %, (*n* = 643)	< 0,001***
*Harninkontinenz*	19,6 %, (*n* = 244)	44,4 %, (*n* = 656)	< 0,001***	50,4 %, (*n* = 369)	34,5 %, (*n* = 287)	< 0,001***
*Mobilitätseinschränkungen*	26,7 %, (*n* = 278)	64,9 %, (*n* = 944)	< 0,001***	72,4 %, (*n* = 521)	52,6 %, (*n* = 423)	< 0,001***
*Sehbeeinträchtigung*	29,4 %, (*n* = 328)	45,9 %, (*n* = 737)	< 0,001***	48,9 %, (*n* = 383)	41,0 %, (*n* = 354)	0,018*
*Subjektive Gedächtnisverschlechterung*	28,1 %, (*n* = 308)	37,0 %, (*n* = 596)	< 0,001***	37,4 %, (*n* = 288)	36,3 %, (*n* = 308)	0,754
*Depressive Symptomatik*	13,1 %, (*n* = 118)	24,4 %, (*n* = 318)	< 0,001***	28,4 %, (*n* = 185)	17,9 %, (*n* = 133)	< 0,001***
*Polypharmazie*	27,9 %, (*n* = 304)	48,7 %, (*n* = 759)	< 0,001***	49,1 %, (*n* = 344)	48,1 %, (*n* = 415)	0,732
*Adipositas*	22,2 %, (*n* = 250)	18,5 %, (*n* = 263)	0,073	20,6 %, (*n* = 136)	15,2 %, (*n* = 127)	0,037*
*Bildungsgruppe (CASMIN)*						
Niedrige	47,4 %, (*n* = 450)	67,2 %, (*n* = 916)	0,004**	70,5 %, (*n* = 472)	62,0 %, (*n* = 444)	< 0,001***
Mittlere	36,0 %, (*n* = 397)	23,7 %, (*n* = 414)		25,0 %, (*n* = 233)	21,7 %, (*n* = 181)	
Hohe	16,5 %, (*n* = 360)	9,1 %, (*n* = 393)		4,6 %, (*n* = 101)	16,4 %, (*n* = 292)	
*Alleinlebend*	24,5 %, (*n* = 284)	48,7 %, (*n* = 681)	< 0,001***	60,5 %, (*n* = 444)	30,0 %, (*n* = 237)	< 0,001***

Signifikanzniveau: *p < 0,05; **p < 0,01; ***p < 0,001

**Abb. 1:**
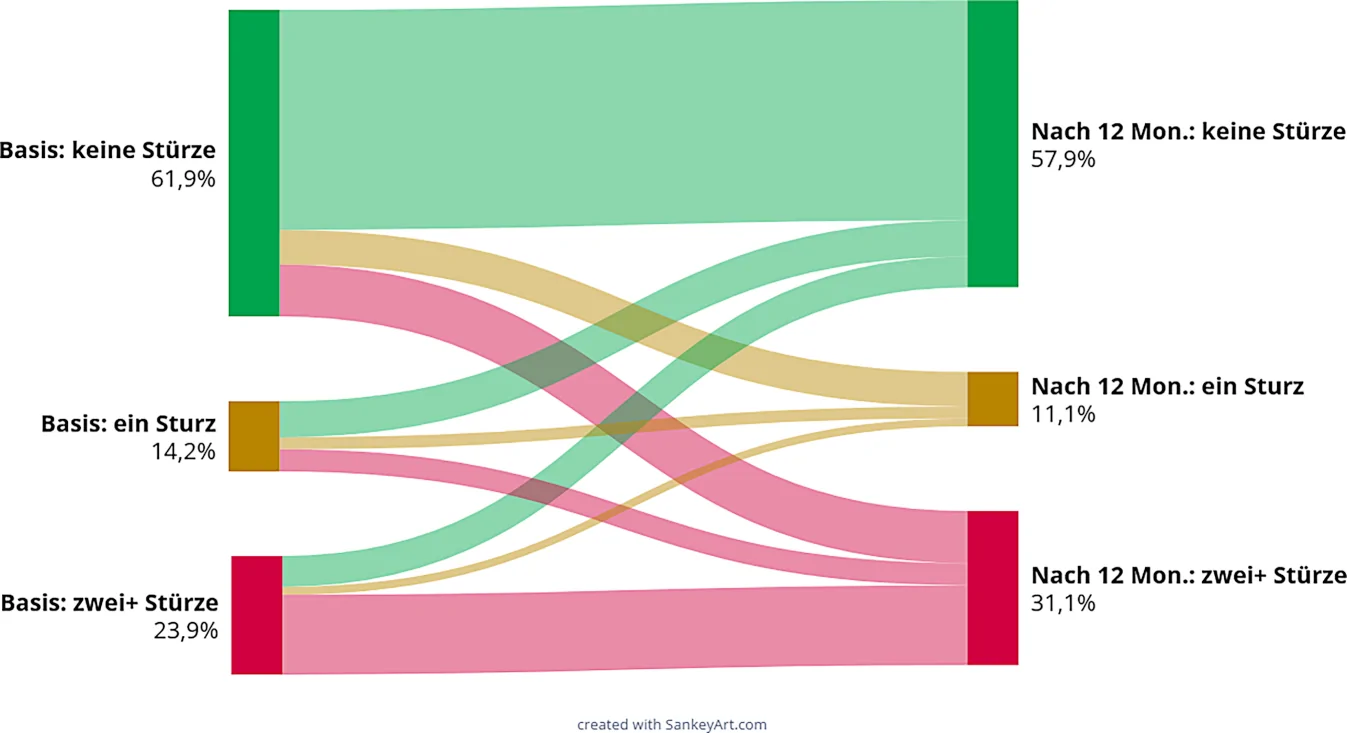
Sturzgeschehen zur Basisbefragung und im darauffolgenden 12-Monats-Zeitraum

Abb. 1 zeigt Veränderungen des Sturzgeschehens zwischen Basis- und Nachbefragung. 9,9 % der zuvor nicht gestürzten Personen berichteten im nachfolgenden 12-Monats-Zeitraum mindestens einen Sturz, während 4,3 % der Personen mit mindestens einem Sturzereignis zur Basisbefragung im Folgejahr keinen Sturz angaben.

### Einfluss von Risikofaktoren auf das Sturzgeschehen

Tab. 2 gibt einen Überblick über die Merkmale der Teilnehmenden ab 80 Jahren und die bivariaten Zusammenhänge mit erhöhtem Sturzrisiko. Die in die Modelle aufgenommenen Faktoren wiesen keine starke Kollinearität auf (Varianzinflation nahe 1). Polypharmazie war signifikant bivariat mit einem erhöhten Sturzrisiko (*p* < 0,001) assoziiert.

**Tab. 2:** Risikofaktoren für Stürze bei Personen ab 80 Jahren nach 12 Monaten (gewichtete %, rohe *n* und *p*-Werte für Gruppenunterschiede)

	Stürze innerhalb 12 Monaten nach Basisbefragung			
	Kein Sturz (53,3 %, *n* = 887)	Ein Sturz (13,1 %; *n* = 245)	2 und mehr Stürze (33,6 %; *n* = 423)	*p*-Wert
*Stürze (zur Basisbefragung)*				
Keine	77,0 %, (*n* = 698)	61,8 %, (*n* = 147)	33,6 %, (*n* = 151)	< 0,001***
Ein Sturz	13,2 %, (*n* = 105)	21,3 %, (*n* = 45)	14,6 %, (*n* = 65)	
2 und mehr Stürze	9,8 %, (*n* = 66)	16,8 %, (*n* = 42)	51,8 %, (*n* = 183)	
*Multimorbidität*	74,5 %, (*n* = 609)	79,3 %, (*n* = 184)	83,2 %, (*n* = 351)	0,030*
*Harninkontinenz*	36,1 %, (*n* = 271)	40,1 %, (*n* = 79)	57,9 %, (*n* = 225)	< 0,001***
*Mobilitätseinschränkungen*	50,4 %, (*n* = 369)	65,6 %, (*n* = 137)	82,3 %, (*n* = 307)	< 0,001***
*Sehbeeinträchtigung*	39,0 %, (*n* = 321)	45,8 %, (*n* = 103)	53,6 %, (*n* = 223)	0,002**
*Subjektive Gedächtnisverschlechterung*	31,3 %, (*n* = 256)	27,6 %, (*n* = 66)	50,1 %, (*n* = 209)	< 0,001***
*Depressive Symptomatik*	14,9 %, (*n* = 100)	12,9 %, (*n* = 28)	38,0 %, (*n* = 123)	< 0,001***
*Polypharmazie*	40,9 %, (*n* = 326)	43,8 %, (*n* = 104)	61,7 %, (*n* = 231)	< 0,001***
*Adipositas*	14,8 %, (*n* = 119)	21,7 %, (*n* = 44)	23,1 %, (*n* = 76)	0,024*
*Bildungsgruppe (CASMIN)*				
Niedrige	63,7 %, (*n* = 428)	66,3 %, (*n* = 120)	69,6 %, (*n* = 242)	0,018*
Mittlere	24,9 %, (*n* = 214)	20,5 %, (*n* = 56)	24,7 %, (*n* = 106)	
Hohe	11,4 %, (*n* = 232)	13,2 %, (*n* = 67)	5,7 %, (*n* = 72)	
*Alleinlebend*	45,4 %, (*n* = 331)	48,9 %, (*n* = 100)	51,9 %, (*n* = 173)	0,225

Signifikanzniveau: *p < 0,05; **p < 0,01; ***p < 0,001

Im adjustierten Modell waren Harninkontinenz, Sehbeeinträchtigung und depressive Symptomatik mit einer höheren Sturzwahrscheinlichkeit verbunden (Tab. 3).

**Tab. 3:** Assoziation zwischen Stürzen, Polypharmazie und weiteren Risikofaktoren^+^: Schätzungen der unadjustierten Odds Ratio (*OR*)/adjustierten Odds Ratio aus verschiedenen Regressionsmodellen

Variable	Unadj. OR (95 %-KI)	*p*-Wert	Adj. OR (95 %-KI)	*p*-Wert
*Geschlecht (ref. männlich)*				
Weiblich	1,28 (0,95–1,72)	0,109	1,06 (0,77–1,44)	0,730
*Polypharmazie (ref. keine)*				
Polypharmazie	1,78 (1,30–2,44)	< 0,001	1,39 (0,99–1,96)	0,059
*Multimorbidität (ref. keine)*				
Multimorbidität	1,64 (1,09–2,46)	0,018	1,09 (0,72–1,67)	0,673
*Harninkontinenz (ref. keine)*				
Harninkontinenz	2,06 (1,49–2,85)	< 0,001	1,65 (1,18–2,32)	0,004*
*Sehbeeinträchtigung (ref. keine)*				
Sehbeeinträchtigung	1,69 (1,23–2,32)	0,001	1,41 (1,02–1,95)	0,039*
*Subjektive Gedächtnisverschlechterung (ref. keine)*				
Subjektive Gedächtnisverschlechterung	1,68 (1,21–2,33)	0,002	1,33 (0,94–1,86)	0,103
*Depressive Symptomatik (ref. keine)*				
Depressive Symptomatik	2,32 (1,53–3,53)	< 0,001	1,61 (1,00–2,6)	0,049*
*Adipositas (ref. keine)*				
Adipositas	1,69 (1,11–2,58)	0,015	1,51 (0,97–2,35)	0,066
*Bildungsgruppe (ref. hohe)*				
Niedrige	1,64 (1,17–2,29)	0,004	1,30 (0,91–1,85)	0,154
Mittlere	1,52 (1,03–2,25)	0,037	1,38 (0,91–2,08)	0,131

*ref.* Referenzkategorie

^+^ Mit Fokus auf durchführbare Präventionsmaßnahmen wurden die Variablen „vorausgegangene Stürze“ und „Mobilitätseinschränkungen“ für die Adjustierungen ausgeschlossen, um die Effekte für die beeinflussbaren Risikofaktoren abzuschätzen

Signifikanzniveau: *p < 0,05; **p < 0,01; ***p < 0,001

### Einnahme von Arzneimitteln, die das Sturzrisiko erhöhen (FRIDs)

Von 742 Personen der Studienpopulation ab 80 Jahren, deren Arzneimittelgebrauch genauer untersucht werden konnte, erhielten 59,2 % mindestens ein FRID. Am häufigsten verordnet wurden Diuretika (37,9 %), gefolgt von Opioiden (11,9 %), Alpha-Blockern bei Prostatahyperplasie (9,0 %), Antidepressiva (8,8 %), Antiepileptika (8,1 %) sowie Vasodilatatoren bei Herzerkrankungen (5,3 %). Die FRID-Einnahmehäufigkeit im Detail zeigt Tab. 4.

**Tab. 4:** Prävalenz der Einnahme von Arzneimitteln, die das Sturzrisiko erhöhen (Fall Risk Increasing Drugs, *FRIDs*) nach Arzneimitteltypen (Personen ab 80 Jahren)

FRIDs* (Arzneimittelklassen (ATC-Code) in Klammern)	Anteil in gewichteten %	Anzahl *n*
Benzodiazepine (N03AE, N05BA, N05CD)	3,2	15
Antipsychotika (N05A)	2,7	18
Benzodiazepin-verwandte Arzneimittel (Z-Drugs; N05CF)	2,3	12
Opioide (N02A)	11,9	70
Antidepressiva (N06A)	8,8	68
Anticholinergika (N04A, R03BB, S01FA)	1,9	12
Antiepileptika (N03A)	8,1	53
Diuretika (C03, C02L)	37,9	249
Alpha-Blocker-Antihypertensiva (C02CA)	1,7	8
Alpha-Blocker bei Prostatahyperplasie (G04CA)	9,0	96
Zentral wirkende Antihypertensiva (C02A)	2,3	13
Antihistaminika (R06A)	2,3	10
Vasodilatatoren bei Herzerkrankungen (C01D)	5,3	30
Arzneimittel bei überaktiver Blase/Dranginkontinenz (G04BD)	3,9	36
*FRIDs (gesamt)*	*59,2*	*432*

* FRIDs-Verwendung ist definiert als die Einnahme von mindestens einem Arzneimittel aus der in der Liste genannten Arzneimitteltypen gemäß der ATC-Klassifikation (Anatomical Therapeutic Chemical). Die Arzneimittelklassen sind nicht überlappend

Nach 12 Monaten berichteten 35,8 % der Teilnehmenden, die keine FRIDs einnahmen, mindestens einen Sturz, während 49,6 % der Teilnehmenden mit FRID mindestens einen Sturz berichteten. FRIDs waren im unadjustierten Modell signifikant mit Stürzen assoziiert, jedoch nicht im adjustierten Modell (Tab. 5).

**Tab. 5:** Assoziation zwischen Stürzen, FRIDs und weiteren Risikofaktoren: Schätzungen der unadjustierten Odds Ratio (*OR*)/adjustierten Odds Ratio aus verschiedenen Regressionsmodellen

Variable	Unadj. OR (95 %-KI)	*p*-Wert	Adj. OR (95 %-KI)	*p*-Wert
*Geschlecht (ref. männlich)*				
Weiblich	1,16 (0,76–1,78)	0,492	1,02 (0,65–1,61)	0,932
*FRIDs (ref. keine)*				
FRIDs	1,93 (1,20–3,10)	0,007	1,59 (0,99–2,56)	0,057
*Multimorbidität (ref. keine)*				
Multimorbidität	2,00 (1,16–3,44)	0,013	1,32 (0,75–2,31)	0,335
*Harninkontinenz (ref. keine)*				
Harninkontinenz	1,79 (1,13–2,82)	0,013	1,38 (0,86–2,23)	0,183
*Sehbeeinträchtigung (ref. keine)*				
Sehbeeinträchtigung	1,85 (1,18–2,91)	0,008	1,55 (0,97–2,49)	0,069
*Subjektive Gedächtnisverschlechterung (ref. keine)*				
Subjektive Gedächtnisverschlechterung	1,71 (1,07–2,74)	0,026	1,48 (0,89–2,46)	0,130
*Depressive Symptomatik (ref. keine)*				
Depressive Symptomatik	2,17 (1,17–4,04)	0,014	1,62 (0,83–3,16)	0,161
*Adipositas (ref. keine)*				
Adipositas	2,29 (1,25–4,21)	0,008	2,30 (1,17–4,56)	0,016*
*Bildungsgruppe (ref. hohe)*				
Niedrige	1,54 (0,93–2,54)	0,094	1,29 (0,73–2,29)	0,377
Mittlere	1,67 (0,96–2,89)	0,070	1,73 (0,95–3,15)	0,075

*ref.* Referenzkategorie

Signifikanzniveau: *p < 0,05; **p < 0,01; ***p < 0,001

## Diskussion

Etwa 4 von 10 Teilnehmenden der Studie Gesundheit 65+, die 80 Jahre oder älter waren, stürzten mindestens einmal in den 12 Monaten vor Studienbeginn und knapp die Hälfte stürzte während des Beobachtungszeitraums von einem Jahr. Europaweite Ergebnisse aus dem Survey of Health, Ageing and Retirement in Europe (SHARE) zeigen eine ähnliche Tendenz; Personen ab 80 Jahren stürzen deutlich häufiger als jüngere Personen [39]. Dieser hohe Anteil, besonders in Anbetracht der steigenden Zahl Hochaltriger in der Bevölkerung, weist darauf hin, dass die Maßnahmen zur Sturzprävention noch nicht hinreichend greifen.

Unsere Ergebnisse zeigen, dass sich – abgesehen von Mobilitätseinschränkungen und vorangegangenen Stürzen – Harninkontinenz, Sehbeeinträchtigung und depressive Symptomatik als signifikante sowie Polypharmazie und FRIDs als weitere Risikofaktoren erwiesen, die Ansatzpunkte für die Prävention liefern. Gerade bei Hochaltrigen könnte daher in der ärztlichen Praxis eine Anamnese erfolgen, die neben der Sturzhistorie auch die verschiedenen Risikofaktoren erfasst. Auf dieser Basis können anschließend adäquate Interventionsmaßnahmen eingeleitet werden. Dies entspricht auch dem Vorgehen, das in den „World guidelines for falls prevention and management for older adults“ dargelegt wird [40]. Besonders vielversprechend erscheint hierbei die Betrachtung der eingenommenen Arzneimittel. Darüber hinaus könnten Menschen, die Pflegeleistungen beziehen, sowie ihre An- und Zugehörigen, auch mit Unterstützung ambulanter Pflegedienste, für die Risikofaktoren sensibilisiert und entsprechend informiert werden, wie es z. B. im Expertenstandard „Sturzprophylaxe in der Pflege“ [41] vorgeschlagen wird.

Polypharmazie zeigte in den unadjustierten Analysen eine deutliche Assoziation mit Stürzen, die nach Adjustierung nicht mehr signifikant war. Dieses Muster deutet darauf hin, dass ein Teil der beobachteten Assoziation zwischen Polypharmazie und Stürzen durch Begleiterkrankungen und funktionelle Einschränkungen erklärt wird. Praktisch unterstreicht dies die Bedeutung regelmäßiger Medikationsüberprüfungen, um Sturzrisiken zu reduzieren.

Auch andere Studien zeigen, dass Polypharmazie ein Risikofaktor für Stürze ist, insbesondere wenn die Medikation FRIDs enthält [25, 42, 43]. Einige Analysen deuten darauf hin, dass die reine Anzahl der eingenommenen Arzneimittel ohne das Vorhandensein von FRIDs keinen unabhängigen Risikofaktor für Stürze darstellt [24]. Unsere Studie zeigte nach Adjustierung, dass weder Polypharmazie noch FRIDs ein primäres Sturzrisiko darstellen, hier ist möglicherweise die zugrunde liegende Morbidität bedeutsamer. Besonderes die Kombination aus Polypharmazie und der Anwendung von Antidepressiva oder Benzodiazepinen ist stark mit einer erhöhten Rate verletzungsbedingter Stürze korreliert [44].

In unserer Studie beobachteten wir zudem, dass depressive Symptomatik mit dem Auftreten von mehr als einem Sturz assoziiert war. Das Sturzrisiko wird im Zusammenhang mit Depressionen möglicherweise durch die Behandlung mit Antidepressiva erhöht [13]. Depression, aber auch andere neurologische Erkrankungen wie eine Parkinson- oder Alzheimer-Erkrankung können die Mobilität einschränken, das Gleichgewicht verschlechtern und dadurch Stürze begünstigen [5]. In Kombination mit einer Behandlung mit Antidepressiva oder anderen FRIDs erhöht sich das Sturzrisiko zusätzlich [13, 28].

Das Risiko steigt bereits deutlich an, wenn ältere Menschen 2 oder mehr FRIDs gleichzeitig einnehmen, was auf kumulative Effekte oder potenziell schädliche Wechselwirkungen hindeutet [45]. Ein zentraler Diskussionspunkt bleibt dabei die Multimorbidität, da chronische Erkrankungen oft sowohl die Ursache für Polypharmazie als auch für eine erhöhte Sturzneigung sind. Ältere Menschen mit Multimorbidität, die gleichzeitig FRIDs einnehmen, weisen ein erhöhtes Sturzrisiko auf. Dieses hängt sowohl mit chronischen Erkrankungen wie Arthrose, Sehverlust und peripheren Nervenstörungen zusammen als auch mit Nebenwirkungen der FRID-Einnahme, etwa Sedierung, Schwindel und posturaler Instabilität [46]. Zur Rolle von Adipositas bei Stürzen beschrieb zudem eine australische Studie die Einnahme von Arzneimitteln als einen Mediator für den Zusammenhang zwischen Adipositas und Stürzen bei älteren Menschen im häuslichen Umfeld [47].

Für diese Studie wurden FRIDs analog zu der STOPPFall-Klassifikation identifiziert [34, 35]. Die Beschränkung auf 3 ATC-Hauptgruppen (N04A, R03BB, S01FA) in der Kategorie der Anticholinergika diente primär der Vermeidung von Doppelzählungen innerhalb des STOPPFall-Tools. Viele Arzneimittel, die ebenfalls starke anticholinerge Eigenschaften besitzen, sind bereits in anderen der insgesamt 14 definierten FRID-Klassen enthalten, wie etwa Antidepressiva (N06A), Antipsychotika (N05A) oder sedierende Antihistaminika (R06). Um die Liste übersichtlich zu gestalten und sicherzustellen, dass ein Wirkstoff nicht in mehreren Kategorien gleichzeitig gewertet wird, werden in der Gruppe der „Anticholinergika“ gezielt nur jene Substanzen aufgeführt, die nicht bereits durch die anderen spezifischen Gruppen abgedeckt sind. Dazu gehören beispielsweise Parkinsonmittel (N04A), inhalative Parasympatholytika (R03BB) oder Mydriatika (S01FA). Klinisch ist jedoch entscheidend, dass das anticholinerge Risiko (Anticholinergic Burden) über alle eingenommenen Medikamentengruppen hinweg summiert wird, da diese Effekte kumulativ das Sturzrisiko erhöhen [48].

Screening-Instrumente wie STOPPFall können Klinikerinnen und Kliniker dabei unterstützen, riskante Wirkstoffklassen gezielt zu identifizieren und die Medikationssicherheit zu verbessern [49]. Trotz dieser theoretischen Vorteile zeigt die aktuelle Evidenzlage, dass das alleinige Absetzen von FRIDs in kontrollierten Studien häufig keine unmittelbare signifikante Reduktion der Sturzhäufigkeit bewirkt [50]. Daher ist ein multifaktorieller Ansatz essenziell, der Medikationsbewertungen mit Kraft- und Balanceübungen sowie einer Anpassung des Wohnumfelds kombiniert [46].

### Stärken und Limitationen

Gesundheit 65+ ist eine bevölkerungsrepräsentative Studie, die besondere Aufmerksamkeit der Rekrutierung von Hochaltrigen gewidmet hat [30]. Durch dieses Studiendesign konnten 1742 Personen ab 80 Jahren in die Studie eingeschlossen werden, es war auch möglich, gesetzliche Vertretungen einzubeziehen. Die Beantwortung der Fragen erfolgte überwiegend über einen Papierfragebogen; die Angaben basieren größtenteils auf Selbstangaben, die abhängig von dem aktuellen Gesundheitszustand der Teilnehmenden sind. Durch die Mehrthemenbefragung ist es möglich, Assoziationen zwischen Stürzen und relevanten Risikofaktoren abzubilden. Es ist allerdings zu berücksichtigen, dass Menschen mit schweren gesundheitlichen Beeinträchtigungen seltener an Studien teilnehmen können und zudem eine erhöhte Wahrscheinlichkeit für einen vorzeitigen Abbruch in Längsschnittstudien aufweisen. Ferner konnten im Rahmen der Studie Sturzfolgen nicht erfasst werden, sodass Verletzungen infolge eines Sturzes nicht dokumentiert sind. Eine weitere Limitation unserer Studie ist, dass die Klassifikation sturzrelevanter Substanzen nur in einer Teilstichprobe erfolgen konnte. Dennoch ergaben sich Hinweise auf das potenzielle Risiko von Arzneimitteln, die diese Substanzen enthalten.

## Fazit

Die hohe Sturzprävalenz bei Personen ab 80 Jahren in Deutschland zeigt, dass bisherige Präventionsmaßnahmen nicht ausreichen. Stürze führen häufig zu Verletzungen, Krankenhausaufenthalten, Autonomieverlust sowie Angst und sozialer Isolation. Wir konnten zeigen, dass neben Mobilitätseinschränkungen und früheren Stürzen Harninkontinenz, Adipositas, Sehbeeinträchtigungen, depressive Symptome, Polypharmazie und insbesondere sturzrisikoerhöhende Medikamente das Risiko erhöhen. Sturzprävention sollte daher multidimensional ansetzen: durch Medikamentenüberprüfungen, Sehkontrollen, Behandlung von Inkontinenz und Depression sowie individuell angepasste Bewegungsprogramme. Nur ein ganzheitlicher Ansatz kann Stürze im hohen Alter langfristig reduzieren und Lebensqualität erhalten.

## Data Availability

Die während der vorliegenden Studie erzeugten und/oder analysierten Datensätze sind nicht öffentlich zugänglich, da die Einwilligung (Informed Consent) der Studienteilnehmenden die öffentliche Bereitstellung der Daten nicht abdeckt. Sie können aber auf begründete Anfrage beim Forschungsdatenzentrum des RKI angefordert werden.
